# Nicotine enhances murine airway contractile responses to kinin receptor agonists via activation of JNK- and PDE4-related intracellular pathways

**DOI:** 10.1186/1465-9921-11-13

**Published:** 2010-01-29

**Authors:** Yuan Xu, Yaping Zhang, Lars-Olaf Cardell

**Affiliations:** 1 Division of Ear, Nose and Throat Diseases, CLINTEC, Karolinska Institutet, Karolinska University Hospital, Huddinge, Sweden

## Abstract

**Background:**

Nicotine plays an important role in cigarette-smoke-associated airway disease. The present study was designed to examine if nicotine could induce airway hyperresponsiveness through kinin receptors, and if so, explore the underlying mechanisms involved.

**Methods:**

Murine tracheal segments were cultured for 1, 2 or 4 days in serum-free DMEM medium in presence of nicotine (1 and 10 μM) or vehicle (DMSO). Contractile responses induced by kinin B_1 _receptor agonist, des-Arg^9^-bradykinin, and B_2 _receptor agonist, bradykinin, were monitored with myographs. The B_1 _and B_2 _receptor mRNA expressions were semi-quantified using real-time PCR and their corresponding protein expressions assessed with confocal-microscopy-based immunohistochemistry. Various pharmacological inhibitors were used for studying intracellular signaling pathways.

**Results:**

Four days of organ culture with nicotine concentration-dependently increased kinin B_1 _and B_2 _receptor-mediated airway contractions, without altering the kinin receptor-mediated relaxations. No such increase was seen at day 1 or day 2. The airway contractile responses to 5-HT, acetylcholine and endothelin receptor agonists remained unaffected by nicotine. Two different neuronal nicotinic receptor antagonists MG624 and hexamethonium blocked the nicotine-induced effects. The enhanced contractile responses were accompanied by increased mRNA and protein expression for both kinin receptors, suggesting the involvement of transcriptional mechanisms. Confocal-microscopy-based immunohistochemistry showed that 4 days of nicotine treatment induced activation (phosphorylation) of c-Jun N-terminal kinase (JNK), but not extracellular signal-regulated kinase 1 and 2 (ERK1/2) and p38. Inhibition of JNK with its specific inhibitor SP600125 abolished the nicotine-induced effects on kinin receptor-mediated contractions and reverted the enhanced receptor mRNA expression. Administration of phosphodiesterase inhibitors (YM976 and theophylline), glucocorticoid (dexamethasone) or adenylcyclase activator (forskolin) suppressed the nicotine-enhanced airway contractile response to des-Arg^9^-bradykinin and bradykinin.

**Conclusions:**

Nicotine induces airway hyperresponsiveness via transcriptional up-regulation of airway kinin B_1 _and B_2 _receptors, an effect mediated via neuronal nicotinic receptors. The underlying molecular mechanisms involve activation of JNK- and PDE4-mediated intracellular inflammatory signal pathways. Our results might be relevant to active and passive smokers suffering from airway hyperresponsiveness, and suggest new therapeutic targets for the treatment of smoke-associated airway disease.

## Introduction

Airway hyperreactivity is a major feature of asthma and a consequence of airway inflammation. It is well-known that both active [1,2] and passive cigarette smoke exposure [3,4] can cause airway hyperresponsiveness (AHR). Maternal cigarette smoking increases the risk for wheezing in early life and the development of childhood asthma [5,6]. Second-hand smoke exposure in asthmatics is associated with poor asthma control, greater asthma severity and greater risk of asthma-related hospital admission [7]. *In vivo *studies in guinea pigs have demonstrated that chronic exposure to tobacco smoke selectively increases airway reactivity to bradykinin and capsaicin, without altering responses to methacholine or histamine [8]. This suggests an important role for bradykinin in tobacco smoke-induced AHR.

Tobacco smoke is a composite of irritant molecules, including nicotine, acetaldehyde, formaldehyde, nitrogen oxides, and heavy metals, and long-term exposure results in chronic airway inflammation, AHR and in some individuals, chronic obstructive pulmonary disease (COPD). Nicotine is one of the more important components of cigarette smoke. It is also widely marketed as an aid to smoke cessation in forms of nicotine-replacement products. Once inhaled, nicotine is quickly taken up by the bloodstream and distributed throughout the body, to act primarily on nicotinic acetylcholine receptors. In humans, functional nicotinic receptors, of both the muscle and neuronal subtypes, are present on fibroblasts and in bronchial epithelial cells. They have the ability to activate protein kinase C as well as members of the mitogen-activated protein kinases (MAPKs) including extracellular signal-regulated kinase 1 and 2 (ERK1/2) and p38 [9]. Many of the detrimental health effects of cigarette-smoke are believed to be due to nicotine's ability to affect the immune system. Stimulation of the nicotinic receptor produces complex reactions including both inflammatory [10] and anti-inflammatory effects [11], including modulation of allergic responses [12]. There is also evidence suggesting that nicotine can directly interfere with the phosphorylation of intracellular inflammatory signal molecules such as c-Jun N-terminal kinase (JNK) and ERK1/2, without involvement of the nicotinic receptors [13]. However, the knowledge about the intracellular mechanisms behind nicotine's effects is still limited.

Inhibition of phosphodiesterases (PDEs) results in the elevation of cyclic AMP (cAMP) and cyclic GMP (cGMP) which lead to a variety of cellular effects including airway smooth muscle relaxation and inhibition of cellular inflammation [14]. The archetypal non-selective PDE inhibitor theophylline shows anti-inflammatory properties and has been used clinically for more than 70 years. However, its narrow therapeutic window and extensive interactions with other drugs limits its clinical use. PDE4 is specific for the break-down of intracellular cAMP and PDE4 inhibitors have been intensely investigated for the treatment of asthma and COPD. The PDE4 subtype PDE4D5 has been recently shown to be the key physiological regulator of beta-adrenergic receptor-induced cAMP turnover within human airway smooth muscle [15]. It is well-known that cells respond to stimuli through a "network" of different signaling pathways. Interestingly, the cAMP pathway can interact with the MAPK cascade. cAMP negatively regulates MAPK p38 activation, and thereby contributing to tumor necrosis factor (TNF)-α-induced apoptosis in certain cell types [16]. Activation of ERK5 and the subsequent transcription of c-JUN, but not ERK1/2, can be blocked by cAMP through cAMP-dependent protein kinase (PKA) [17].

Airway G-protein coupled receptors (GPCR), such as kinin, 5-hydroxytryptamine (5-HT), endothelin and muscarinic acetylcholine receptors, not only mediate airway smooth muscle contraction, but also airway inflammation and remodelling [18]. We have previously, by using an *in vitro *model of chronic airway inflammation, demonstrated that cytokines can induce transcriptional up-regulation of kinin B_1 _and B_2 _receptors and subsequently increase kinin receptor-mediated contractions [19]. Our receptor characterization studies using specific pharmacological antagonists have demonstrated that the B_1 _receptor is selectively activated by des-Arg^9^-bradykinin, whereas the B_2 _receptor is activated by bradykinin [20]. The B_2 _receptor is constitutively expressed in airways, while the B_1 _receptor is inducible following tissue injury and inflammation [21]. Stimulation of the kinin receptors in airways causes both bronchoconstriction and epithelium-dependent relaxation, as well as mucus secretion, edema and cough. The relaxation is mediated via activation of cyclooxygenase (COX) and release of the bronchodilator prostaglandin E_2 _(PGE_2_) [21]. The mechanism behind AHR to kinins appears to involve activation of intracellular MAPKs and the down-stream transcription factor nuclear factor-kappaB (NF-κB) [20,22].

One of the hypotheses of the present study is that long-term exposure to nicotine can induce activation of airway MAPK-mediated inflammatory signal pathways and subsequently cause AHR via up-regulation of kinin receptors. This idea is based on previous data revealing activation of MAPK-mediated NF-κB inflammatory signal pathways in AHR along with an up-regulation of kinin receptors [20,22,23]. This is further corroborated by *in vivo *studies showing selective up-regulation of kinin receptors after exposure to cigarette smoke [8] and by *in vitro *results presenting activation of MAPK in human bronchial cells following stimulation of nicotinic receptors [9].

Reports of a role for PDE4 inhibitors in asthma and COPD treatment [14] together with the known interactions between the MAPK and cAMP pathways [16,17] lead to our interest for possible nicotine-induced changes in PDE4 and cAMP pathway. Thus, the present study was designed to investigate if long-term exposure to nicotine could induce AHR to bradykinin and des-Arg^9^-bradykinin through the selective up-regulation of kinin receptors and to explore the underlying intracellular inflammatory signal transduction mechanisms involved, with focus on both MAPK and PDE4.

## Materials and methods

### Tissue preparation

Male BALB/c J mice (9-10 weeks old) were sacrificed by cervical dislocation. The whole trachea was rapidly removed and placed into cold Dulbecco's modified Eagle's medium (DMEM; 4500 mg L^-1 ^D-glucose, 110 mg L^-1 ^sodium pyruvate, 584 mg L^-1 ^L-glutamine). For *in vitro *pharmacology and immunohistochemistry studies, the trachea was cut into ring segments, each containing three cartilage rings, while the whole trachea was kept intact for real-time PCR studies. The experimental protocol was approved by the local Ethics Committee.

### Organ culture

The tracheal rings, alternatively the whole trachea, were placed individually in wells of a 96- or 24-well plate (Ultra-low attachment; Sigma, St. Louis, MO, U.S.A.) with 300 μL or 1 mL serum-free DMEM culture medium supplemented with penicillin (100 U mL^-1^) and streptomycin (100 μg mL^-1^). All tissue were incubated at 37°C in humidified 5% CO_2 _in air with either nicotine (1 or 10 μM), vehicle (dimethyl sulfoxide, DMSO, 0.1%) or nicotine (10 μM) plus various inhibitors for 1, 2 or 4 days. The segments were transferred to new wells containing fresh medium with supplements of nicotine, vehicle or inhibitors every day.

### In-vitro pharmacology

The cultured tracheal ring was immersed in temperature-controlled (37°C) myograph bath (Organ Bath Model 700 MO, J.P. Trading, Aarhus, Denmark) containing 5 ml Krebs-Henseleit buffer solution (143 mM Na^+^, 5.9 mM K^+^, 1.5 mM Ca^2+^, 2.5 mM Mg^2+^, 128 mM Cl^-^, 1.2 mM H_2_PO_4_^2-^, 1.2 mM SO_4_^2-^, 25 mM HCO^3- ^and 10 mM D-glucose), continuously equilibrated with 5% CO_2 _in 95% O_2 _at a pH of 7.4. Each tracheal segment was mounted on two L-shaped metal prongs. One of the prongs was connected to a force-displacement transducer for continuous recording of isometric tension by Chart software (ADInstruments Ltd, Hastings, U.K.), while the other prong was a displacement device, allowing gentle stretching of the tracheal rings mounted. A basal tension of 0.8 mN was gradually reached over the course of at least 90 min. The segment viabilities were tested using 60 mM KCl. KCl was later washed out with Kreb-Henseleit buffer solution for three times until the segments reached basal tension. Thereafter, each segment was incubated with 3 μM indomethacin for 30 min before administration of agonists to inhibit epithelium-dependent relaxations. Agonists were then administered cumulatively to produce their concentration-effect curves. To test their relaxant properties, segments were first pre-constricted with 1 μM carbachol, and after reaching stable plateaus, the concentration-effect curves for bradykinin- and des-Arg^9^-bradykinin-induced relaxations were produced in the absence of indomethacin.

### Real-time quantitative PCR

After homogenization of the tissues, the total RNA was extracted using the RNeasy Mini kit following the supplier's instructions (QIAGEN GmbH, Hilden, Germany). The purity of total RNA was checked with a spectrophotometer and the wavelength absorption ratio (260/280 nm) was between 1.7 and 2.0 in all preparations. Reverse transcription of total RNA (0.3-0.4 μg) to cDNA was carried out using Omniscript™ reverse transcriptase kit (QIAGEN GmbH, Hilden, Germany) in 20 μl volume reaction at 37°C for 1 h using Mastercycler personal PCR machine (Eppendorf AG, Hamburg, Germany).

Specific primers for murine kinin B_1 _and B_2 _receptors, and the house keeping gene glyceraldehyde-3-phosphate dehydrogenase (GAPDH) were designed using Prime Express 2.0 software (Applied Biosystem, Forster city, CA, USA) and synthesized with DNA Technology A/S (Aarhus, Denmark). The sequences are as following:

Kinin B_1 _receptor [Accession Number: NM_007539]: Forward: 5'-CCA TAG CAG AAA TCT ACC TGG CTA AC-3'; Reverse: 5'-GCC AGT TGA AAC GGT TCC-3'

Kinin B_2 _receptor [Accession Number: NM_009747]: Forward: 5'-ATG TTC AAC GTC ACC ACA CAA GTC-3'; Reverse: 5'-TGG ATG GCA TTG AGC CAA C-3'

GAPDH [Accession Number: XM_001473623]: Forward: 5'-CAT GGC CTT CCG TGT TCC TA-3'; Reverse: 5'-TGC TTC ACC ACC TTC TTG ATG-3'

Real-time PCR was performed with QuantiTect™ SYBR^® ^Green PCR kit (QIAGEN GmbH, Hilden, Germany) in the Smart Cycler^® ^II system (Cepheid, Sunnyvale, CA, USA). The system automatically monitors the binding of a fluorescent dye SYBR^® ^Green to double-stranded DNA during each cycle of PCR amplification. The real-time PCR was prepared in 25 μl reaction volumes and carried out with heating 95°C for 15 min followed by touchdown PCR i.e. denature at 94°C for 30 sec and annealing at 66°C for 1 min for the first PCR cycle, thereafter, a 2°C decrease for the annealing temperature in every cycle until 56°C. Finally, 40 thermal cycles with 94°C for 30 sec and 55°C for 1 min were performed. The data were analyzed with the threshold cycle (C_T_) method and the specificity of the PCR products was checked by the dissociation curves. A blank (no template) was included in all the experiments as negative control. The relative amount of mRNA was expressed as the C_T _values of mRNA for kinin B_1 _or B_2 _receptor in relation to the C_T _values for the house-keeping gene GAPDH in the same sample.

### Immunohistochemistry with confocal microscopy

After organ culture, the tracheal segments were immersed in a fixative solution consisting of 4% paraformaldehyde in 0.1 M phosphate buffer (pH 7.4) for 3 h at 4°C. After fixation, the specimens were dehydrated in 20% sucrose in 0.1 M phosphate buffer (pH 7.4) for 24 h at 4°C, then frozen in Tissue-Tek (Sakura Finetek Europe B.V., Zoeterwoude, Netherlands) and stored at -80°C. Sections were cut to 10-μm-thick slices in a cryostat and mounted on SuperFrost Plus slides (Menzel GMBH & COKG, Braunschweig, Germany).

Immunohistochemistry were carried out using standard protocols, i.e. the sections were incubated with the primary antibody overnight at 4°C and the secondary antibody for 1 h at room temperature in darkness. Primary and secondary antibodies as well as the dilutions used were as following: kinin B_1 _receptor (1:50, goat, Santa Cruz Biotechnology, Inc. Santa Cruz, CA, USA), kinin B_2 _receptor (1:100, rabbit, Alexis Biochemical, Lausen, Switzerland), phospho-SAPK/JNK (Thr183/Tyr185) (1:50, rabbit, Cell Signalling Technology, Inc. Beverly, MA, USA), phospho-p38 MAPK (Thr180/Tyr182) (1:100, rabbit, Cell Signalling Technology) and phospho-ERK1/2 MAPK (Thr202/Tyr204) (1:100, rabbit, Cell Signalling Technology). The appropriate secondary antibodies, goat anti-rabbit IgG H&L conjugated to fluorescein isothiocynate (FITC) or Texas Red or Alexa Fluor^® ^488 donkey anti-goat IgG H&L was used for fluorescence microscopic imaging, respectively. In the control experiments, either the primary antibody or the secondary antibody was omitted. The stained specimens were examined under a confocal microscope (Nikon, C1plus, Nikon Instruments Inc., NY, USA). The fluorescence intensity was measured and analysed by Image J software http://rsb.info.nih.gov/ij.

To avoid systemic errors, the nicotine-treated specimen and the corresponding control are always cultured, fixated, stained, examined and scanned at the same time as the same batch, and the setting of the confocal microscope is kept unchanged throughout. This ensures comparability between the groups. The measurements are repeated for each specimen at 6 preset randomly selected sections, at each section the florescence density was measured at 6 areas, and the mean florescence density was obtained from 6 experiments. All measurements are checked and confirmed by another senior researcher.

### Reagents

Bradykinin, des-Arg^9^-bradykinin, sarafotoxin 6b and sarafotoxin 6c were purchased from Neosystem S.A., Strasbourg, France. SP600125 (anthrax(1,9-cd)pyrazol-6(2H)-one) was from Calbiochem, Bad Soden, Germany. Nicotine, dexamethasone, indomethacin, 5-HT, carbachol, acetylcholine, YM976, theophylline, forskolin, hexamethonium, MG624, DMEM and Krebs-Henseleit buffer were from Sigma, St. Louis, MO, U.S.A. The stock solutions of bradykinin, des-Arg^9^-bradykinin, sarafotoxin 6b and sarafotoxin 6c were prepared in 0.1% bovine serum albumin. Nicotine, YM976, SP600125, MG624 and forskolin were dissolved in DMSO. Theophylline, hexamethonium, 5-HT, carbachol and acetylcholine were dissolved in distilled water, and indomethacin in 95% ethanol. All agonists were serially diluted with physiological saline prior to experiments.

### Data analysis

All data were expressed as mean ± S.E.M. Agonist concentration-effect curve data from individual segments were fitted to the Hill equation using an iterative, least-squares method (GraphPad Prism 5, San Diego, CA, U.S.A.) to provide estimates of maximal contraction (E_max_) and pEC_50 _(negative logarithm of the agonist concentration that produces half of its maximal effect). Contractile responses to agonists are all expressed in mN. Concentration-effect curves obtained from myograph studies were compared using two-way analysis of variance (ANOVA) with Bonferroni's post-test. Unpaired student's *t*-test with Welch's correction was used when two groups were compared. P ≤ 0.05 was considered to be statistically significant.

## Results

### Effects of nicotine on kinin B_1 _and B_2 _receptor-mediated airway contractions

**Figure 1:**
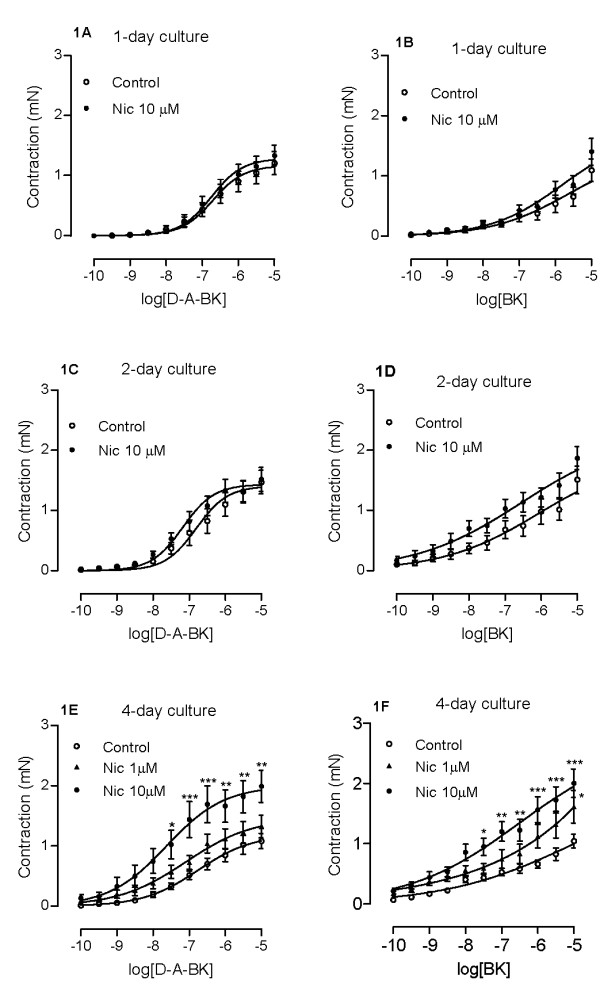
**Nicotine-induced effects on kinin receptor-mediated airway contractions**. Tracheal segments were cultured for 1 day (A, B), 2 days (C, D) or 4 days (E, F) in presence of vehicle (Control, 0.1% DMSO) or nicotine (Nic, 1 or 10 μM). Contractions were induced by des-Arg^9^-bradykinin (D-A-BK; A, C, E) or bradykinin (BK; B, D, F). Each data point is derived from 15-22 experiments and data is presented as mean ± S.E.M. Statistical analysis was performed using two-way ANOVA with Bonferroni's post-test. Control vs Nic. * P < 0.05; ** P < 0.01; *** P < 0.001.

In order to assess the time-course of nicotine effects on the airway contraction, tracheal segments were organ-cultured for 1, 2 or 4 days in the presence of nicotine (10 μM) or vehicle. A tendency towards an increased airway contractile response to des-Arg^9^-bradykinin and bradykinin was seen already after 2 days of nicotine treatment and this increase reached statistical significance at day 4 (Fig. 1A-F, Table 1).

**Table 1:** Effects of nicotine on des-Arg^9^-bradykinin- and bradykinin-induced airway contractions

Incubation time	Nicotine (μM)		des-Arg^9^-bradykinin			Bradykinin	
		***n***	E_max _(mN)	pEC_50_	***n***	E_max _(mN)	pEC_50_
Day 1	0 (Ctrl)	17	1.21 ± 0.19	6.49 ± 0.12	15	0.99 ± 0.18	5.81 ± 0.13
	10	18	1.33 ± 0.17	6.52 ± 0.11	16	1.29 ± 0.16	5.79 ± 0.18
Day 2	0 (Ctrl)	16	1.47 ± 0.19	6.56 ± 0.14	17	1.51 ± 0.23	6.15 ± 0.27
	10	16	1.52 ± 0.19	6.94 ± 0.13	17	1.86 ± 0.19	6.75 ± 0.35
Day 4	0 (Ctrl)	18	1.16 ± 0.13	6.96 ± 0.19	21	1.40 ± 0.20	6.72 ± 0.38
	1	16	1.89 ± 0.26	6.28 ± 0.50	19	2.10 ± 0.34	6.57 ± 0.36
	10	21	2.04 ± 0.25 **	7.20 ± 0.20	22	2.18 ± 0.26 *	7.30 ± 0.25

Tracheal segments were cultured for 1, 2 or 4 days in presence of vehicle (0.1% DMSO, Ctrl) or nicotine (1 or 10 μM). E_max _and pEC_50 _for des-Arg^9^-bradykinin and bradykinin are presented as mean ± S.E.M. Statistical analysis was performed using unpaired student's *t*-test with Welch's correction. Nicotine vs. Ctrl (DMSO). * P < 0.05, **P < 0.01. n = number of experiments performed.

Concentration-effects of nicotine were tested on the tracheal segments after 4-day culture. A lower nicotine concentration (1 μM) did not significantly increase contractile responses to des-Arg^9^-bradykinin and bradykinin. Culture with 10 μM of nicotine significantly increased the E_max _for both agonists. Although a tendency towards an increased pEC_50 _can be seen, it did not reach statistical significance (Fig. 1E-F, Table 1). Nicotine (1 or 10 μM) treatment for 1, 2 or 4 days did not affect the contractile response mediated by 5-HT, cholinergic (Table 2) or endothelin receptors (Table 3).

**Table 2:** Effects of nicotine on 5-HT- and acetylcholine-induced airway contractions

Incubation time	Nicotine (μM)		5-HT			Acetylcholine	
		***n***	E_max _(mN)	pEC_50_	***n***	E_max _(mN)	pEC_50_
Day 1	0 (Ctrl)	9	1.87 ± 0.32	6.47 ± 0.13	8	5.81 ± 0.74	6.51 ± 0.12
	10	10	1.97 ± 0.26	6.45 ± 0.10	8	6.20 ± 0.62	6.46 ± 0.07
Day 2	0 (Ctrl)	11	2.01 ± 0.29	6.83 ± 0.09	8	6.45 ± 0.70	6.57 ± 0.06
	10	12	1.99 ± 0.31	6.87 ± 0.09	8	5.95 ± 0.73	6.56 ± 0.10
Day 4	0 (Ctrl)	10	2.01 ± 0.23	6.98 ± 0.08	6	6.04 ± 1.05	6.43 ± 0.07
	1	9	1.89 ± 0.28	7.00 ± 0.13	6	5.24 ± 0.64	6.56 ± 0.12
	10	8	1.88 ± 0.18	6.89 ± 0.18	6	5.70 ± 0.49	6.61 ± 0.11

Tracheal segments were cultured for 1, 2 or 4 days in presence of vehicle (0.1% DMSO, Ctrl) or nicotine (1 or 10 μM). E_max _and pEC_50 _for 5-HT and acetylcholine are presented as mean ± S.E.M. Statistical analysis was performed using unpaired student's *t*-test with Welch's correction. Nicotine vs Ctrl (DMSO). No significant differences were found between the two groups. n = number of experiments performed.

**Table 3:** Effects of nicotine on endothelin receptor-mediated airway contractions

Incubation time	Nicotine (μM)		ET_A_			ET_B_	
		***n***	E_max _(mN)	pEC_50_	***n***	E_max _(mN)	pEC_50_
Day 1	0 (Ctrl)	10	3.61 ± 0.40	7.52 ± 0.14	9	3.49 ± 0.68	8.00 ± 0.13
	10	10	3.40 ± 0.33	7.50 ± 0.07	10	3.52 ± 0.53	7.89 ± 0.07
Day 2	0 (Ctrl)	4	3.74 ± 0.87	7.40 ± 0.20	4	3.33 ± 0.34	8.24 ± 0.13
	10	4	4.22 ± 0.85	7.52 ± 0.11	4	3.15 ± 0.60	8.00 ± 0.13
Day 4	0 (Ctrl)	9	4.32 ± 0.71	7.81 ± 0.10	9	4.31 ± 0.73	8.05 ± 0.09
	1	9	4.13 ± 0.42	7.74 ± 0.07	9	4.03 ± 0.46	8.17 ± 0.13
	10	8	4.67 ± 0.37	7.86 ± 0.10	8	4.47 ± 0.38	8.18 ± 0.12

Tracheal segments were cultured for 1, 2 or 4 days in presence of vehicle (0.1% DMSO, Ctrl) or nicotine (1 or 10 μM). ET_A_: endothelin receptor type A; ET_B_: endothelin receptor type B. Responses of ET_B _receptors were tested with the selective ET_B _agonist sarafotoxin 6c, while responses to ET_A _receptors were tested with the non-selective ET-receptor agonist sarafotoxin 6b after the desensitization of ET_B _receptors [39]. E_max _and pEC_50 _are presented as mean ± S.E.M. Statistical analysis was performed using unpaired student's *t*-test with Welch's correction. Nicotine vs Ctrl (DMSO). No significant differences were found between the two groups. n = number of experiments performed.

### Effects of nicotine on kinin B_1 _and B_2 _receptor-mediated airway relaxations

**Figure 2:**
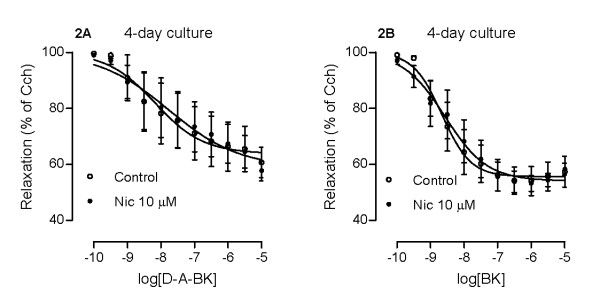
**Nicotine-induced effects on kinin receptor-mediated airway relaxations**. Tracheal segments were cultured for 4 days in presence of vehicle (Control, 0.1% DMSO) or nicotine (Nic, 10 μM). Relaxations were induced by des-Arg^9^-bradykinin (D-A-BK; A) or bradykinin (BK; B) after pre-constriction with carbachol (1 μM). Each data point is derived from 6-8 experiments and data is presented as mean ± S.E.M. Statistical analysis was performed using two-way ANOVA with Bonferroni's post-test. Control vs Nic. No significant differences were found between the two groups.

Bradykinin and des-Arg^9^-bradykinin can also produce relaxant effects on preconstricted tracheal segments. This relaxation is dependent on the airway epithelium as well as on COX activity and EP receptors [21]. Pretreatment of the segments with COX-inhibitor indomethacin for 30 min makes it possible to study receptor-mediated contractions, as described in Figure 1. Absence of indomethacin allows characterization of kinin-induced relaxations succeeding pre-contraction of the segments with carbachol (1 μM). After 4 days of organ culture with nicotine (10 μM) or vehicle (0.1% DMSO), neither B_1 _nor B_2 _receptor-mediated relaxations are affected by nicotine (Fig. 2A-B).

### Effects of nicotinic receptor antagonists on nicotine-enhanced kinin B_1 _and B_2 _receptor-mediated airway contractions

**Figure 3:**
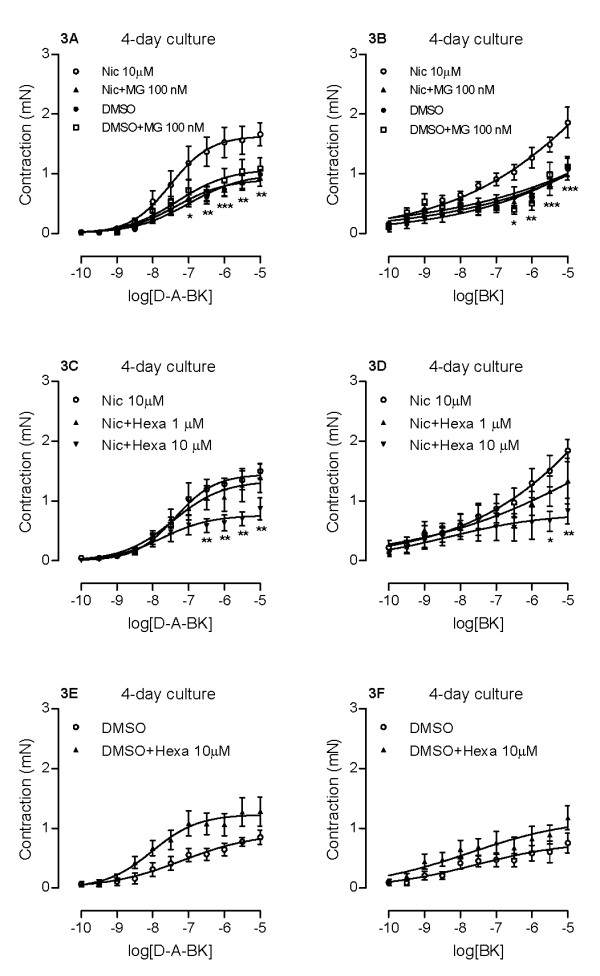
**Effects of neuronal nicotinic receptor antagonists on nicotine-enhanced kinin B_1 _and B_2 _receptor-mediated contractions**. Tracheal segments were cultured for 4 days in presence of vehicle (DMSO, 0.1%) or nicotine (Nic, 10 μM) with/without neuronal nicotinic receptor antagonist MG624 (MG, 100 nM, A, B) or hexamethonium (Hexa, 1 or 10 μM, C-F). Contractions were induced by des-Arg^9^-bradykinin (D-A-BK; A, C, E) or bradykinin (BK; B, D, F). Each data point is derived from 3-6 experiments and data is presented as mean ± S.E.M. Statistical analysis was performed using two-way ANOVA with Bonferroni's post-test. Nic vs Nic+MG/Hexa (A-D), DMSO vs DMSO+MG/Hexa (A, B, E, F). * P < 0.05; ** P < 0.01; *** P < 0.001.

Neuronal nicotinic acetylcholine receptors can very roughly be divided into two groups: α-bungarotoxin-sensitive receptors that contain the α7 subunit and α-bungarotoxin-insensitive receptors. MG624 is a specific antagonist for the α7 subunit [24], while hexamethonium inhibits α-bungarotoxin-insensitive receptors [25]. In order to find out if the observed nicotine effects on B_1 _and B_2 _receptor-mediated contractions are mediated through nicotinic receptors, tracheal segments were cultured with 10 μM nicotine in combination with either MG624 (100 nM) or hexamethonium (1 or 10 μM). Results show that MG624 completely revoked the enhanced contractions caused by nicotine for both kinin receptors without altering the contractile response in the control group (0.1% DMSO) at all (Fig. 3A-B). In analogy, hexamethonium (10 μM) also depressed the nicotine-enhanced kinin effects (Fig. 3C-D). Applying the same hexamethonium concentration to the DMSO-treated control segments did not cause a decrease in contractile responses for B_1 _and B_2 _receptors, but rather a weak tendency towards increased contraction (Fig. 3E-F). Altogether, the results suggest a clear involvement of neuronal nicotinic receptors in nicotine-induced effects on B_1 _and B_2 _receptor-mediated contractions in airways.

### Effects of nicotine on airway kinin B_1 _and B_2 _receptor mRNA and protein expressions

**Figure 4:**
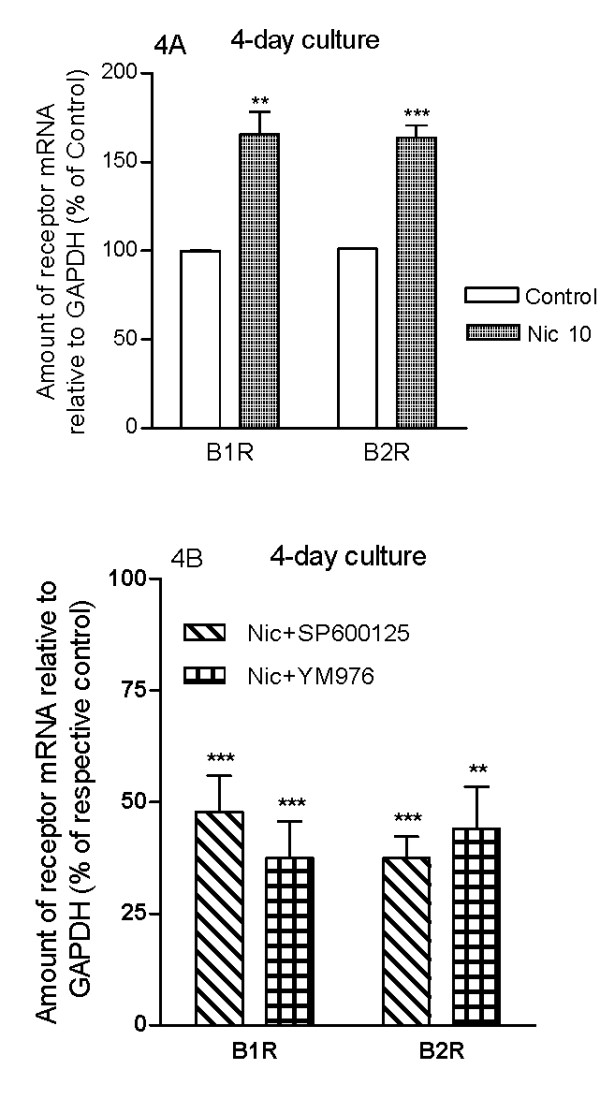
**Kinin B_1 _(B1R) and B_2 _(B2R) receptor mRNA expression**. Tracheal segments were cultured for 4 days in presence of vehicle (DMSO, control) or nicotine (Nic, 10 μM) (A). JNK inhibitor SP600125 or PDE4 inhibitor YM976 was added to 4-day culture with nicotine (10 μM) (B). Each data point is derived from 3-6 experiments and data is presented as mean ± S.E.M. Statistical analysis was performed using unpaired student's *t*-test with Welch's correction. Control vs Nic (A); Nic vs Nic+SP600125/YM976 (B). ** P < 0.01; *** P < 0.001.

**Figure 5:**
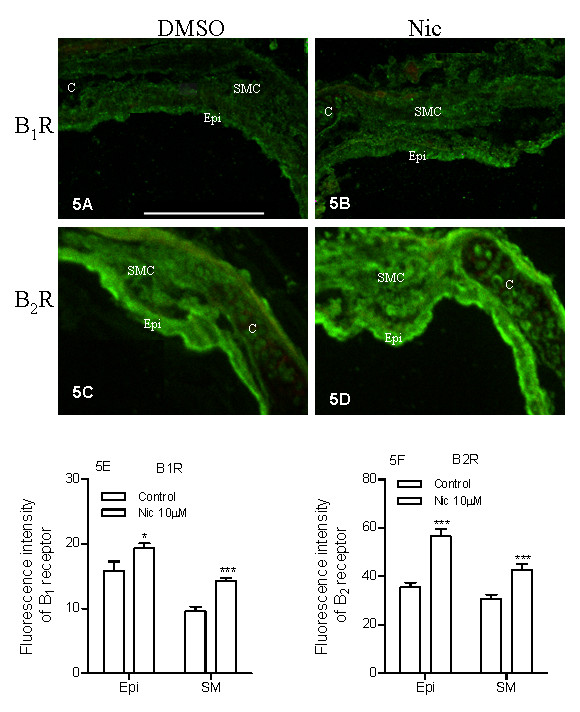
**Nicotine-induced effects on kinin B_1 _(B1R) and B_2 _(B2R) receptor protein expression**. Tracheal segments were cultured for 4 days in presence of vehicle (DMSO, A, C) or nicotine (Nic, 10 μM, B, D). The reference bar corresponds to 25 μm. The intensity of fluorescence was semi-quantified using Image J software (E, F). Epi = epithelium; SMC = smooth muscle cells; and C = cartilage. Each data point is derived from 6 experiments. Two-tailed unpaired Student's *t*-test with Welch's correction was preformed. Control vs Nic. * P < 0.05; *** P < 0.001.

The relative amount of mRNA for kinin B_1 _and B_2 _receptors was quantified by real-time PCR. Four days of organ culture in the presence of nicotine (10 μM) increased the mRNA expression for both receptors, compared to control (Fig. 4A). The corresponding protein expression was examined using confocal-microscopy-based immunohistochemistry. An increase in kinin B_1 _(Fig. 5A-B) and B_2 _(Fig. 5C-D) receptor protein expressions were seen in both the airway epithelial and smooth muscle cells (Fig. 5E-F). In the control segments, the expression of B_1 _receptors is higher in the epithelial cells compared to the smooth muscle cells; while after nicotine treatment, the increase in B_1 _receptor protein expression was more prominent in the smooth muscle cells than in the epithelial cells (Fig. 5E). For B_2 _receptors, their expressions in the control segments are similar between epithelial cells and smooth muscle cells; while after nicotine treatment, B_2 _receptors are expressed more in the epithelial cells than the smooth muscle cells (Fig. 5F).

### Intracellular MAPK signal transduction mechanism studies

**Figure 6:**
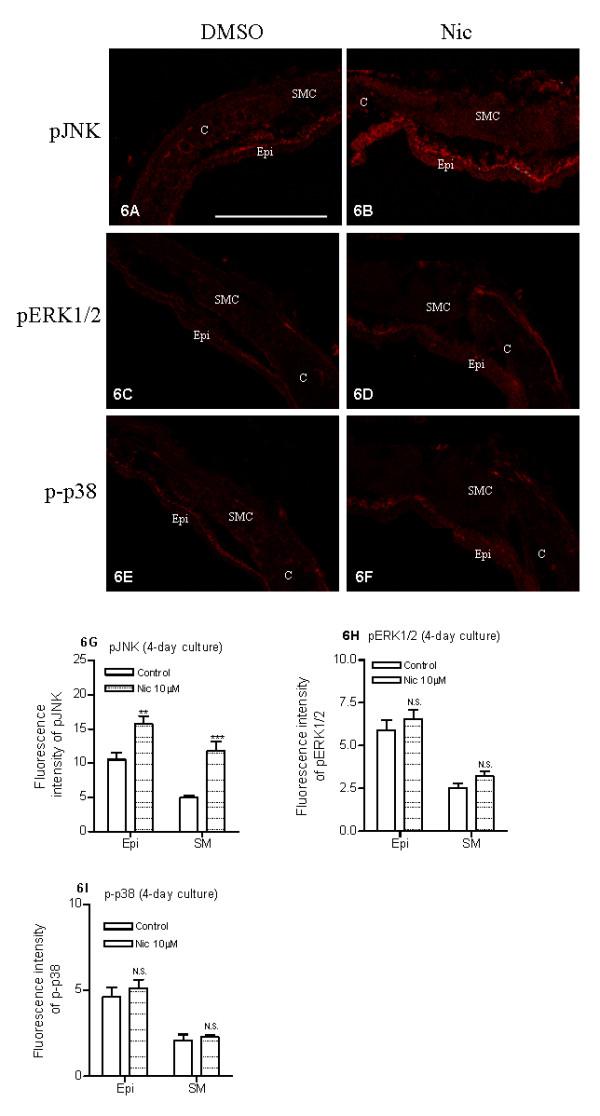
**Nicotine-induced effects on phosphorylated JNK (pJNK), ERK1/2 (pERK1/2) and p38 (p-p38) protein expression**. Tracheal segments were cultured for 4 days in presence of vehicle (DMSO, A, C, E) or nicotine (Nic, 10 μM, B, D, F). The reference bar corresponds to 25 μm. The intensity of fluorescence was semi-quantified by Image J software (G, H, I). Epi = epithelium; SMC = smooth muscle cells; and C = cartilage. Each data point is derived from 6 experiments. Two-tailed unpaired Student's *t*-test with Welch's correction was preformed. Control vs Nic. ** P < 0.01; *** P < 0.001, N.S. = no significance.

To explore the underlying intracellular signal transduction mechanisms behind the reported nicotine effects on airway kinin receptors, the activation (phosphorylation) of JNK, ERK1/2 and p38 signal molecules were studied with confocal-microscopy-based immunohistochemistry. After 4 days of organ culture with nicotine (10 μM), an activation of JNK was observed in the airway epithelial and in smooth muscle cells compared to control (Fig. 6A-B). This increase was most marked in the smooth muscle cells (Fig. 6G). In the control segments, the expression of phosphorylated ERK1/2 (Fig. 6C) and p38 (Fig. 6E) was more abundant in the tracheal epithelium than smooth muscle cells (Fig. 6H-I). However, in contrast to JNK, no significant differences in ERK1/2 (Fig. 6C, D, H) or p38 (Fig. 6E, F, I) activities were found between the specimen treated with nicotine (10 μM) for 4 days and the control (DMSO).

**Figure 7:**
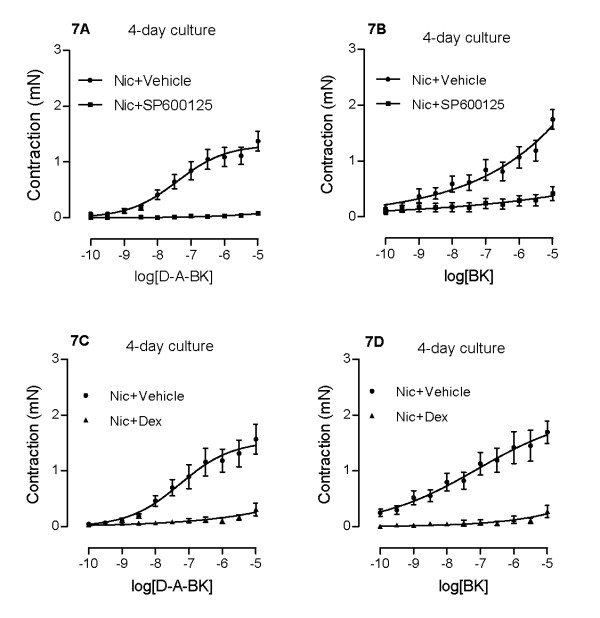
**Effects of JNK inhibitor SP600125 (10 μM) and dexamethasone (Dex, 1 μM) on nicotine-enhanced des-Arg^9^-bradykinin- (A, C) and bradykinin- (B, D) induced contractions**. Tracheal segments were cultured for 4 days in presence of nicotine (Nic, 10 μM) together with vehicle or SP600125 (A, B) or dexamethasone (C, D). Each data point is derived from 10-12 experiments.

In order to link the activation of JNK to nicotine-induced up-regulation of kinin B_1 _and B_2 _receptors, a specific JNK inhibitor SP600125 (10 μM) was added together with nicotine during the 4 days of culture. Pharmacological inhibition of JNK abolished the nicotine-enhanced kinin B_1 _and B_2 _receptor-mediated contractions (Fig. 7A-B) and decreased the nicotine-enhanced kinin B_1 _and B_2 _receptor mRNA expressions (Fig. 4B).

### Effects of dexamethasone and PDE inhibition

Dexamethasone is a potent glucocorticoid and well-known anti-inflammatory drug. Administration of dexamethasone (1 μM) together with nicotine in the organ culture for 4 days almost completely abolished the nicotine-enhanced airway contractions to both des-Arg^9^-bradykinin (Fig. 7C) and bradykinin (Fig. 7D).

**Figure 8:**
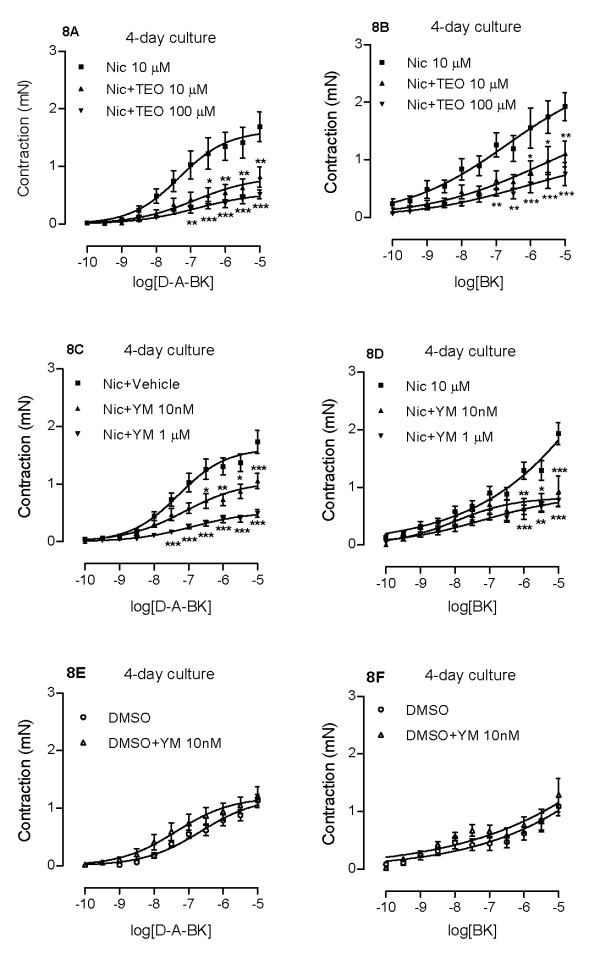
**Effects of YM976 (C, D, E, F) and theophylline (A, B) on nicotine-enhanced des-Arg^9^-bradykinin- (A, C, E) and bradykinin- (B, D, F) induced contractions**. Tracheal segments were cultured for 4 days in absence (E, F) or presence (A-D) of nicotine (Nic, 10 μM) together with vehicle, YM976 (YM, 1 μM or 10 nM) or theophylline (TEO, 10 or 100 μM). Each data point is derived from 4-17 experiments and presented as mean ± S.E.M. Statistical analysis was performed using two-way ANOVA with Bonferroni's post-test. Nic vs Nic+TEO (A, B); Nic+vehicle vs Nic+YM (C, D); DMSO vs DMSO+YM (E, F). * P < 0.05; ** P < 0.01; *** P < 0.001.

To explore the role of PDE in nicotine-enhanced contractile response to the kinins, PDE inhibitors YM976 and theophylline were applied. Theophylline is a non-selective PDE inhibitor, while YM976 is a specific inhibitor for PDE4. The latter PDE subtype is specific for cAMP and thought to be of importance for asthmatic inflammation [26]. After 4 days of treatment with the PDE inhibitors, YM976 concentration-dependently attenuated nicotine up-regulated B_1 _receptor-mediated contractions (Fig. 8C), whereas the dose-relation was less obvious for contractions mediated via B_2 _receptors (Fig. 8D). Contractile responses of the control (DMSO) segments were unaffected by YM976 (Fig. 8E-F). The decrease in receptor-mediated contractions is paralleled with a significant decrease in nicotine-enhanced kinin B_1 _and B_2 _receptor mRNA expression shown by real-time PCR (Fig. 4B). Theophylline exhibited similar effects as YM976, effectively attenuating both B_1 _and B_2 _receptor-mediated airway contractions. The theophylline effect is clearly concentration-dependent (Fig. 8A-B).

### Effects of cAMP

**Figure 9:**
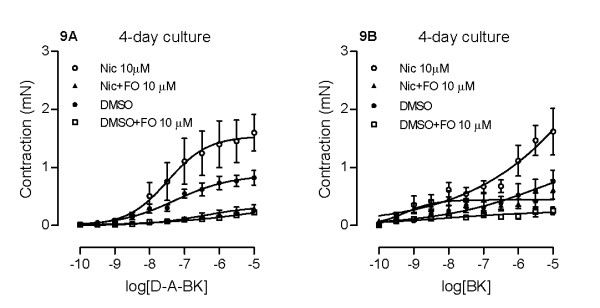
**Effects of forskolin on nicotine-enhanced des-Arg^9^-bradykinin- (A) and bradykinin- (B) induced contractions**. Tracheal segments were cultured for 4 days in absence (DMSO) or presence of nicotine (Nic, 10 μM) together with vehicle or forskolin (FO, 10 μM). Each data point is derived from 3-4 experiments and presented as mean ± S.E.M.

Forskolin is an adenylyl-cyclase activator and raises the level of intracellular cAMP. YM976 inhibits PDE4, the enzyme responsible for the breakdown of cAMP, which in turn also causes an increase in intracellular cAMP levels. To test whether elevation of intracellular cAMP levels is responsible for the PDE inhibitors' ability to attenuate nicotine-enhanced B_1 _and B_2 _receptor-mediated contraction, we treated the segments with forskolin (10 μM) for 4 days in the absence or presence of nicotine (10 μM). Results show that forskolin suppresses contractions induced by both bradykinin and des-Arg^9^-bradykinin, and this is regardless of the presence or absence of nicotine (Fig. 9A-B).

## Discussion

Cigarette smoke is associated with chronic airway inflammation, AHR, increased asthma severity and to a certain degree, asthma development in children [1-7]. Chronic exposure to tobacco smoke increases AHR to bradykinin *in vivo *[8]. The presented study demonstrated for the first time that long-term exposure (for 4 days) of mouse tracheal segments to nicotine causes a concentration-dependent increase of kinin B_1 _and B_2 _receptor-mediated airway contractions. Since B_1 _and B_2 _receptor-mediated relaxation remained unaffected, the resulting netto effect is an increase in contraction. Short-term nicotine exposure (for 1 - 2 days) induced no significant effects. Neither did nicotine treatment affect airway contractions mediated by 5-HT, cholinergic or endothelin receptors. The increase in maximal contraction, without significant change of pEC_50_, seen after 4 days of nicotine treatment suggests an increase in kinin receptor protein expression rather than alteration of receptor sensitivity. This conclusion is further supported by the discovery of an up-regulated protein expression for both B_1 _and B_2 _receptors using confocal microscopy. In addition, real-time PCR reveals a parallel increase in B_1 _and B_2 _receptor mRNA suggesting the involvement of transcriptional mechanisms in nicotine's effects. The neuronal nicotinic receptor antagonists MG624 and hexamethonium both abolish the nicotine-enhanced kinin effect, signifying the participation of nicotinic receptors in the start of the process. Further, the intracellular cascade related to the kinin receptor up-regulation seems to involve JNK- and PDE4-related intracellular signal pathways.

Neuronal nicotinic receptors in non-neuronal cells have been proposed to be mediators of tobacco toxicity since they are considered to have a "hormone-like" function [27]. Our results show that the neuronal nicotinic receptor antagonists MG624 [24] and hexamethonium [25] both inhibit nicotine's effects on the kinin receptor-mediated contractions, without suppressing contractions in control segments. In human smokers, nicotine is not only found in blood plasma, but also in saliva and induced sputum. The nicotine concentrations in saliva can be up to 8 μM during "smoking days" [28] and 5 min after smoking a cigarette, the induced sputum (not contaminated with saliva) contains a surprising 34 μM of nicotine [29]. Therefore, the lungs and bronchial surfaces of smokers might be exposed to a much higher nicotine concentration than that measured in the bloodstream. The concentration that was demonstrated to cause a significant effect in the present study was 10 μM. The same concentration has previously been shown to cause phosphorylation of the MAPK p44/42, an effect that can be inhibited by nAChR antagonists [30]. Similar nicotine concentrations is also known to induce alteration in the gene-expression of macrophage-like human cell line [31].

Many GPCRs are involved in the regulation of the contractile state of airway smooth muscle, including 5-HT, bradykinin, endothelin (type A and type B) and M3 muscarinic acetylcholine receptors. Bradykinin, endothelin and M3 muscarinic receptors are Gq-coupled while 5-HT receptors are Gi-coupled [18]. The presented results show that nicotine up-regulated kinin B_1 _and B_2 _receptor-mediated airway contractions, leaving 5-HT, cholinergic and endothelin receptor-mediated contractions completely unaffected. This suggests that nicotine acts on specific targets within the airways. Thus, the effects observed are neither the result of a general hyperresponsiveness nor due to alteration of down-stream G-protein signaling processes. This idea is further strengthen by our findings of a simultaneous up-regulation of receptor function, mRNA and protein expression. It is known that bradykinin acts as a potent bronchoconstrictor in asthmatic patients, but has no effect in normal individuals [21]. Many studies have also demonstrated a strong link between allergic inflammation, AHR and bradykinin [32-34]. Further, polymorphism in the B_2 _receptor gene has been found to be associated with asthma prior to the age of 4 [35]. Our results support the importance of bradykinin in AHR and reveal a special role for bradykinin in nicotine- and/or tobacco smoke-induced AHR.

Stimulation of the kinin receptors can cause both bronchoconstriction and epithelium-dependent relaxations in the airways. It is interesting to note that though kinin receptor protein expression was increased both on the epithelium and smooth muscle, bradykinin- and des-Arg^9^-bradykinin-induced relaxations were unaffected. This might be due to involvement of different pathways. Stimulation of kinin B_1 _and B_2 _receptors on the airway smooth muscle directly activates the inositol 1,4,5-trisphosphate (IP_3_) pathway increasing intracellular Ca^2+ ^levels which subsequently activates the cellular contractile machinery [18]. Kinin receptor-mediated relaxation, on the other hand, is epithelium-dependent. Bradykinin and des-Arg^9^-bradykinin activate COX and stimulate the release of PGE_2 _from airway epithelial cells which induce airway relaxation through EP receptor activation [21]. Therefore, kinin receptor-mediated relaxations are strongly dependent on intact epithelial functions. Nicotine can damage airway epithelial cells with changes in ionic relations and cause submucosal edema as shown with electron microscopy examination of nicotine-treated rat trachea [36]. This might impair the relaxant functions of airways, disregarding the abundance of kinin receptors.

JNK, ERK1/2 and p38 are the classical members of the MAPK family. They are known to play key roles in the regulation of gene expressions. A recent study with human lung macrophages revealed an increase in MAPK phosphorylation and activation of the MAPK/AP-1 pathway caused by cigarette smoke [37]. In another study of human bronchial epithelial cells, ERK1/2, JNK, but not p38 was strongly activated after treatment with nicotine [13]. A special role of JNK in the pathogenesis of asthma has also been implicated [38]. In the present study, nicotine induced activation of JNK, but not ERK1/2 and p38. SP600125 is a small molecular inhibitor for JNK. At the concentration of 10 μM, SP600125 selectively inhibits the phosphorylation of JNK, but not ERK1/2 or p38 in vessels [39]. Our results show that SP600125 abolished the nicotine-enhanced kinin receptor-mediated contractions and the receptor mRNA expression. These results are well in line with a previous study which has demonstrated that SP600125 exhibits powerful inhibitory effect on TNF-α induced up-regulation of kinin B_1 _and B_2 _receptors in airways [20]. Both bradykinin and des-Arg^9^-bradykinin elicits only negligible contractile responses in fresh segments and the culture procedure *per se *causes an up-regulation of the kinin receptors [20]. The B_1 _and B_2 _receptor-mediated contractions were nearly completely abolished by SP600125, suppressing the contractile response to a level similar to that seen in fresh segments [20]. This suggests that both nicotine and the organ culture procedure induce activation of the same intracellular pathway i.e. the MAPK JNK pathway. The increase in B_1 _and B_2 _receptor mRNA and protein expression after organ culture with nicotine strengthens the evidence for an alteration at the receptor level rather than a down-stream process. Furthermore, SP600125 up to 30 μM causes no alteration in carbachol-elicited contractile responses [20], which excludes the possibility of toxic effects of SP600125 on the contractile machinery of the tracheal segments.

Dexamethasone reduces inflammation and hyperreactivity in asthmatic airways [40,41], inhibits kinin receptor expression in cultured human airway fibroblast and smooth muscle cells [42,43]. It also suppress both TNF-α- and organ culture-induced kinin receptor expression in airway smooth muscle [22]. In line with this, the present data demonstrates that dexamethasone inhibited nicotine-enhanced kinin B_1 _and B_2 _receptor-mediated effects in murine airways. It is interesting to note that the effect of dexamethasone appears to be very similar to those of SP600125. Dexamethasone is classically thought to exert its effects via the inhibition of the pro-inflammatory transcription factors activator protein-1 (AP-1) and NF-κB [44]. The JNK cascade has long been related to the transcription factor NF-κB [45] and its ability to bind to AP-1 and form the transcription complex c-JUN/AP-1 is well-known. Nicotine has been reported to activate NF-κB through phosphorylation of JNK [46]. In addition, cigarette smoke can activate AP-1 also via the MAPK/JNK pathway [37,47]. It is therefore tempting to assume that the presently seen effects of dexamethasone are related to inhibition of transcription factor activation downstream of the JNK pathway. However, it has been recently shown that dexamethasone's intracellular actions are much more complex. They include both inhibition of the upstream negative regulator of JNK and p38 MAPKs called MAP kinase phosphatase-1 [48] and post-transcriptional/translational regulation of gene expressions [42,49].

YM976 is a selective PDE4 inhibitor shown to possess powerful anti-inflammatory and direct broncho-relaxant effects in combination with low emetogenicity [26]. The latter is a common problem with older PDE4 inhibitors. Theophylline is a classical, archetypal, non-specific PDE inhibitor. Both drugs attenuated the enhancement caused by nicotine on kinin B_1 _and B_2 _receptor-mediated airway contractions. Moreover, YM976 also suppresses nicotine-enhanced kinin receptor mRNA expression. PDE4 is expressed in airway smooth muscle cells and increases intracellular concentration of the second messenger cAMP [50]. Inhibition of PDE4 suppresses endotoxin-induced airway inflammation and hyperreactivity [51], inhibits reactive oxygen species production, cell adhesion molecule expression and the release of cytokines from activated T-helper cells, airway epithelial cells, basophils, monocytes and macrophages [52]. The mechanisms behind the effects of PDE inhibitors might be related to changes in cAMP-dependent inflammatory pathways via a reduction of TNF-α-induced expression of RANTES, chemokines and eotaxin in the airway smooth muscle cells [53]. When intracellular cAMP levels were directly raised with the adenylyl cylase activator forskolin, we observed effects similar to those of PDE-inhibitors. The downstream protein kinase PKA has also been reported to be involved in cytokine-stimulated up-regulation of kinin B_2 _receptors [42]. However, inhibition of PDE4 produces a specific depression of nicotine's effects without altering control, while forskolin depresses contractile responses in both the nicotine and control group. This suggests that the nicotine-induced changes might be PDE4-specific. PDE4 is dependent on cAMP to produce a cellular response. Hence, the ability to enhance intracellular cAMP levels, although disparate for both YM976 and theophylline, might be responsible for their suppression of nicotine-enhanced kinin receptor-mediated contractions.

Our results show the simultaneous involvement of both JNK and PDE4/cAMP-mediated pathway in nicotine's effects on kinin receptors. Supporting this, there have been many reports on the "cross-talk" between cAMP and JNK pathway. cAMP has the ability to inhibit JNK activation in human airway smooth muscle cells [54], and in rat renal mesangial cells. Forskolin inhibits MAPK [55]. Activation of ERK5 and the subsequent transcription of c-JUN, but not ERK1/2, can be blocked by cAMP through PKA [17].

To conclude, nicotine has been shown to have the ability to enhance bradykinin- and des-Arg^9^-bradykinin-induced airway contractions without affecting their relaxations. The nicotine effect is mediated by activation of airway neuronal nicotinic receptors which results in a transcriptional up-regulation of kinin B_1 _and B_2 _receptors. The whole process depends on the activation of JNK- and PDE4-related intracellular signal pathways Thus, our findings might provide new therapeutic targets for future treatment of tobacco smoke-associated AHR.

## Competing interests

The authors declare that they have no competing interests.

## Authors' contributions

YX carried out the experiments and data analysis, participated in design of the study and drafted the manuscript. YZ conceived and designed the study, helped with performing the immunohistological studies and drafting the manuscript. LOC supervised the work and provided intellectual input in the study. All authors contributed in writing the manuscript and approved the final version.

## References

[B1] Trigg CJ Bennett JB Tooley M Sibbald B D'Souza MF Davies RJ A general practice based survey of bronchial hyperresponsiveness and its relation to symptoms, sex, age, atopy, and smoking Thorax 1990 45 11 866 872 10.1136/thx.45.11.866 2256016 PMC462785

[B2] Higgins BG Britton JR Chinn S Lai KK Burney PG Tattersfield AE Factors affecting peak expiratory flow variability and bronchial reactivity in a random population sample Thorax 1993 48 9 899 905 10.1136/thx.48.9.899 8236072 PMC464774

[B3] Menon P Rando RJ Stankus RP Salvaggio JE Lehrer SB Passive cigarette smoke-challenge studies, increase in bronchial hyperreactivity J Allergy Clin Immunol 1992 89 2 560 566 10.1016/0091-6749(92)90323-T 1740586

[B4] Janson C Chinn S Jarvis D Zock JP Toren K Burney P Effect of passive smoking on respiratory symptoms, bronchial responsiveness, lung function, and total serum IgE in the European Community Respiratory Health Survey, a cross-sectional study Lancet 2001 358 9299 2103 2109 10.1016/S0140-6736(01)07214-2 11784622

[B5] Strachan D Cook D Health effects of passive smoking. 6. Parental smoking and childhood asthma, longitudinal and case-control studies Thorax 1998 53 3 204 212 10.1136/thx.53.3.204 9659358 PMC1745164

[B6] Illi S von Mutius E Lau S Nickel R Niggemann B Sommerfeld C Wahn U The pattern of atopic sensitization is associated with the development of asthma in childhood J Allergy Clin Immunol 2001 108 5 709 714 10.1067/mai.2001.118786 11692093

[B7] Eisner MD Klein J Hammond SK Koren G Lactao G Iribarren C Directly measured second hand smoke exposure and asthma health outcomes Thorax 2005 60 10 814 821 10.1136/thx.2004.037283 16192366 PMC1747192

[B8] Bergren DR Chronic tobacco smoke exposure increases airway sensitivity to capsaicin in awake guinea pigs J Appl Physiol 2001 90 2 695 704 11160071 10.1152/jappl.2001.90.2.695

[B9] Carlisle DL Hopkins TM Gaither-Davis A Silhanek MJ Luketich JD Christie NA Siegfried JM Nicotine signals through muscle-type and neuronal nicotinic acetylcholine receptors in both human bronchial epithelial cells and airway fibroblasts Respir Res 2004 5 27 10.1186/1465-9921-5-27 15588326 PMC544394

[B10] Vassallo R Kroening PR Parambil J Kita H Nicotine and oxidative cigarette smoke constituents induce immune-modulatory and pro-inflammatory dendritic cell responses Mol Immunol 2008 45 12 3321 3329 10.1016/j.molimm.2008.04.014 18533267 PMC2857673

[B11] Wang H Yu M Ochani M Amella CA Tanovic M Susarla S Li JH Wang H Yang H Ulloa L Nicotinic acetylcholine receptor alpha7 subunit is an essential regulator of inflammation Nature 2003 421 6921 384 388 10.1038/nature01339 12508119

[B12] Mishra NC Rir-Sima-Ah J Langley RJ Singh SP Pena-Philippides JC Koga T Razani-Boroujerdi S Hutt J Campen M Kim KC Nicotine primarily suppresses lung Th2 but not goblet cell and muscle cell responses to allergens J Immunol 2008 180 11 7655 7663 18490768 10.4049/jimmunol.180.11.7655 PMC2614131

[B13] Tsai JR Chong IW Chen CC Lin SR Sheu CC Hwang JJ Mitogen-activated protein kinase pathway was significantly activated in human bronchial epithelial cells by nicotine DNA Cell Biol 2006 25 5 312 322 10.1089/dna.2006.25.312 16716121

[B14] Fan Chung K Phosphodiesterase inhibitors in airways disease Eur J Pharmacol 2006 533 1-3 110 117 10.1016/j.ejphar.2005.12.059 16458289

[B15] Billington CK Le Jeune IR Young KW Hall IP A major functional role for phosphodiesterase 4D5 in human airway smooth muscle cells Am J Respir Cell Mol Biol 2008 38 1 1 7 10.1165/rcmb.2007-0171OC 17673687

[B16] Zhang J Bui T Xiang J Lin A Cyclic AMP inhibits p38 activation via CREB-induced dynein light chain Mol Cell Biol 2006 26 4 1223 1234 10.1128/MCB.26.4.1223-1234.2006 16449637 PMC1367190

[B17] Pearson GW Earnest S Cobb MH Cyclic AMP selectively uncouples mitogen-activated protein kinase cascades from activating signals Mol Cell Biol 2006 26 8 3039 3047 10.1128/MCB.26.8.3039-3047.2006 16581779 PMC1446939

[B18] Billington CK Penn RB Signaling and regulation of G protein-coupled receptors in airway smooth muscle Respir Res 2003 4 2 10.1186/rr195 12648290 PMC152647

[B19] Zhang Y Adner M Cardell LO IL-1beta-induced transcriptional up-regulation of bradykinin B1 and B2 receptors in murine airways Am J Respir Cell Mol Biol 2007 36 6 697 705 10.1165/rcmb.2005-0369OC 17255557

[B20] Zhang Y Adner M Cardell LO Up-regulation of bradykinin receptors in a murine in-vitro model of chronic airway inflammation Eur J Pharmacol 2004 489 1-2 117 126 10.1016/j.ejphar.2004.02.033 15063163

[B21] Barnes PJ Bradykinin and asthma Thorax 1992 47 11 979 983 10.1136/thx.47.11.979 1465760 PMC464122

[B22] Zhang Y Adner M Cardell LO Glucocorticoids suppress transcriptional up-regulation of bradykinin receptors in a murine in vitro model of chronic airway inflammation Clin Exp Allergy 2005 35 4 531 538 10.1111/j.1365-2222.2005.02207.x 15836764

[B23] Bachar O Adner M Uddman R Cardell LO Toll-like receptor stimulation induces airway hyper-responsiveness to bradykinin, an effect mediated by JNK and NF-kappa B signaling pathways Eur J Immunol 2004 34 4 1196 1207 10.1002/eji.200324569 15048731

[B24] Gotti C Balestra B Moretti M Rovati GE Maggi L Rossoni G Berti F Villa L Pallavicini M Clementi F 4-Oxystilbene compounds are selective ligands for neuronal nicotinic alphaBungarotoxin receptors Br J Pharmacol 1998 124 6 1197 1206 10.1038/sj.bjp.0701957 9720791 PMC1565512

[B25] Lawson CJ Homewood J Taylor AJ The Effects of L-glucose on memory in mice are modulated by peripherally acting cholinergic drugs Neurobiol Learn Mem 2002 77 1 17 28 10.1006/nlme.2000.4001 11749083

[B26] Aoki M Kobayashi M Ishikawa J Saita Y Terai Y Takayama K Miyata K Yamada T A novel phosphodiesterase type 4 inhibitor, YM976 (4-(3-chlorophenyl)-1, 7-diethylpyrido[2,3-d]pyrimidin-2(1H)-one), with little emetogenic activity J Pharmacol Exp Ther 2000 295 1 255 260 10991987

[B27] Conti-Fine BM Navaneetham D Lei S Maus AD Neuronal nicotinic receptors in non-neuronal cells, new mediators of tobacco toxicity? Eur J Pharmacol 2000 393 1-3 279 294 10.1016/S0014-2999(00)00036-4 10771024

[B28] Lindell G Farnebo LO Chen D Nexo E Rask Madsen J Bukhave K Graffner H Acute effects of smoking during modified sham feeding in duodenal ulcer patients. An analysis of nicotine, acid secretion, gastrin, catecholamines, epidermal growth factor, prostaglandin E2, and bile acids Scand J Gastroenterol 1993 28 6 487 494 10.3109/00365529309098254 8322024

[B29] Clunes LA Bridges A Alexis N Tarran R In vivo versus in vitro airway surface liquid nicotine levels following cigarette smoke exposure J Anal Toxicol 2008 32 3 201 207 18397571 10.1093/jat/32.3.201 PMC2994604

[B30] Carlisle DL Liu X Hopkins TM Swick MC Dhir R Siegfried JM Nicotine activates cell-signaling pathways through muscle-type and neuronal nicotinic acetylcholine receptors in non-small cell lung cancer cells Pulm Pharmacol Ther 2007 20 6 629 641 10.1016/j.pupt.2006.07.001 17015027

[B31] Koshi R Sugano N Orii H Fukuda T Ito K Microarray analysis of nicotine-induced changes in gene expression in a macrophage-like human cell line J Periodontal Res 2007 42 6 518 526 10.1111/j.1600-0765.2007.00976.x 17956464

[B32] Berman AR Togias AG Skloot G Proud D Allergen-induced hyperresponsiveness to bradykinin is more pronounced than that to methacholine J Appl Physiol 1995 78 5 1844 1852 7649921 10.1152/jappl.1995.78.5.1844

[B33] Eric J Gabra BH Sirois P Implication of the bradykinin receptors in antigen-induced pulmonary inflammation in mice Br J Pharmacol 2003 138 8 1589 1597 10.1038/sj.bjp.0705207 12721115 PMC1573809

[B34] Huang TJ Haddad EB Fox AJ Salmon M Jones C Burgess G Chung KF Contribution of bradykinin B(1) and B(2) receptors in allergen-induced bronchial hyperresponsiveness Am J Respir Crit Care Med 1999 160 5 Pt 1 1717 1723 10556146 10.1164/ajrccm.160.5.9901029

[B35] Kusser B Braun A Praun M Illi S von Mutius E Roscher AA Polymorphisms in the bradykinin B2 receptor gene and childhood asthma Biol Chem 2001 382 5 885 889 10.1515/BC.2001.110 11517947

[B36] Roomans G Vanthanouvong V Dragomir A Kozlova I Wróblewski R Effects of nicotine on intestinal and respiratory epithelium J Submicrosc Cytol Pathol 2002 34 4 381 388 12575837

[B37] Birrell MA Wong S Catley MC Belvisi MG Impact of tobacco-smoke on key signaling pathways in the innate immune response in lung macrophages J Cell Physiol 2008 214 1 27 37 10.1002/jcp.21158 17541958

[B38] Adcock IM Chung KF Caramori G Ito K Kinase inhibitors and airway inflammation Eur J Pharmacol 2006 533 1-3 118 132 10.1016/j.ejphar.2005.12.054 16469308

[B39] Xu CB Zheng JP Zhang W Zhang Y Edvinsson L Lipid-soluble smoke particles upregulate vascular smooth muscle ETB receptors via activation of mitogen-activating protein kinases and NF-kappaB pathways Toxicol Sci 2008 106 2 546 555 10.1093/toxsci/kfn173 18718921

[B40] De Bie JJ Hessel EM Van Ark I Van Esch B Hofman G Nijkamp FP Van Oosterhout AJ Effect of dexamethasone and endogenous corticosterone on airway hyperresponsiveness and eosinophilia in the mouse Br J Pharmacol 1996 119 7 1484 1490 8968559 10.1111/j.1476-5381.1996.tb16062.x PMC1915832

[B41] Trifilieff A El-Hashim A Bertrand C Time course of inflammatory and remodeling events in a murine model of asthma, effect of steroid treatment Am J Physiol Lung Cell Mol Physiol 2000 279 6 L1120 1128 11076802 10.1152/ajplung.2000.279.6.L1120

[B42] Haddad EB Fox AJ Rousell J Burgess G McIntyre P Barnes PJ Chung KF Post-transcriptional regulation of bradykinin B1 and B2 receptor gene expression in human lung fibroblasts by tumor necrosis factor-alpha: modulation by dexamethasone Mol Pharmacol 2000 57 6 1123 1131 10825382

[B43] Schmidlin F Scherrer D Landry Y Gies JP Glucocorticoids inhibit the bradykinin B2 receptor increase induced by interleukin-1beta in human bronchial smooth muscle cells Eur J Pharmacol 1998 354 1 R7 8 10.1016/S0014-2999(98)00478-6 9726641

[B44] Barnes PJ Therapeutic strategies for allergic diseases Nature 1999 402 6760 Suppl B31 38 10.1038/35037026 10586893

[B45] Schulze-Osthoff K Ferrari D Riehemann K Wesselborg S Regulation of NF-kappa B activation by MAP kinase cascades Immunobiology 1997 198 1-3 35 49 9442376 10.1016/s0171-2985(97)80025-3

[B46] Barr J Sharma CS Sarkar S Wise K Dong L Periyakaruppan A Ramesh GT Nicotine induces oxidative stress and activates nuclear transcription factor kappa B in rat mesencephalic cells Mol Cell Biochem 2007 297 1-2 93 99 10.1007/s11010-006-9333-1 17021677 PMC2758082

[B47] Gensch E Gallup M Sucher A Li D Gebremichael A Lemjabbar H Mengistab A Dasari V Hotchkiss J Harkema J Tobacco smoke control of mucin production in lung cells requires oxygen radicals AP-1 and JNK J Biol Chem 2004 279 37 39085 39093 10.1074/jbc.M406866200 15262961

[B48] Zhou Y Ling EA Dheen ST Dexamethasone suppresses monocyte chemoattractant protein-1 production via mitogen activated protein kinase phosphatase-1 dependent inhibition of Jun N-terminal kinase and p38 mitogen-activated protein kinase in activated rat microglia J Neurochem 2007 102 3 667 678 10.1111/j.1471-4159.2007.04535.x 17403137

[B49] Bergmann MW Staples KJ Smith SJ Barnes PJ Newton R Glucocorticoid inhibition of granulocyte macrophage-colony-stimulating factor from T cells is independent of control by nuclear factor-kappaB and conserved lymphokine element 0 Am J Respir Cell Mol Biol 2004 30 4 555 563 10.1165/rcmb.2003-0295OC 14527927

[B50] Torphy TJ Undem BJ Cieslinski LB Luttmann MA Reeves ML Hay DW Identification, characterization and functional role of phosphodiesterase isozymes in human airway smooth muscle J Pharmacol Exp Ther 1993 265 3 1213 1223 8389856

[B51] Toward TJ Broadley KJ Chronic lipopolysaccharide exposure on airway function, cell infiltration, and nitric oxide generation in conscious guinea pigs: effect of rolipram and dexamethasone J Pharmacol Exp Ther 2001 298 1 298 306 11408555

[B52] Sanz MJ Cortijo J Morcillo EJ PDE4 inhibitors as new anti-inflammatory drugs: effects on cell trafficking and cell adhesion molecules expression Pharmacol Ther 2005 106 3 269 297 10.1016/j.pharmthera.2004.12.001 15922015

[B53] Ammit AJ Hoffman RK Amrani Y Lazaar AL Hay DW Torphy TJ Penn RB Panettieri RA Jr Tumor necrosis factor-alpha-induced secretion of RANTES and interleukin-6 from human airway smooth-muscle cells. Modulation by cyclic adenosine monophosphate Am J Respir Cell Mol Biol 2000 23 6 794 802 11104733 10.1165/ajrcmb.23.6.4184

[B54] Kaur M Holden NS Wilson SM Sukkar MB Chung KF Barnes PJ Newton R Giembycz MA Effect of beta2-adrenoceptor agonists and other cAMP-elevating agents on inflammatory gene expression in human ASM cells: a role for protein kinase A Am J Physiol Lung Cell Mol Physiol 2008 295 3 L505 514 10.1152/ajplung.00046.2008 18586957

[B55] Li X Zarinetchi F Schrier RW Nemenoff RA Inhibition of MAP kinase by prostaglandin E2 and forskolin in rat renal mesangial cells Am J Physiol 1995 269 4 Pt 1 C986 991 7485469 10.1152/ajpcell.1995.269.4.C986

